# Giant Prolactin-Secreting Pituitary Adenoma: A Case Report and Literature Review

**DOI:** 10.7759/cureus.85126

**Published:** 2025-05-31

**Authors:** Jorge A Ocon Rodríguez, Angélica López Méndez, Emilio Mondragón Rosas, Airam A Arias Villaverde, Elizabeth Gloria Méndez, Michelle Cruz Méndez, Lourdes Rivas Ayala, José Emiliano González Flores

**Affiliations:** 1 Neurosurgery, Legaria Pediatric Hospital and General Hospital of Mexico, Mexico City, MEX; 2 Pediatric Nephrology, Legaria Pediatric Hospital and National Institute of Pediatrics, Mexico City, MEX; 3 Medicine and Health Sciences, Tecnológico de Monterrey Mexico City Campus, Mexico City, MEX; 4 Pediatrics, Panamerican University, Mexico City, MEX; 5 Medicine, Tecnológico de Monterrey Mexico City Campus, Mexico City, MEX

**Keywords:** cabergoline resistance, craniotomy, dopamine agonists, hyperprolactinemia, men1 mutation, pituitary neoplasms, prolactinoma

## Abstract

Prolactinomas are the most common functional pituitary adenomas in the pediatric population, though they remain rare overall. Male adolescents often present with larger and more aggressive tumors than female adolescents, with delayed symptoms such as visual disturbances, headaches, and hypogonadism due to the absence of early hormonal signs. We report the case of a 15-year-old previously healthy male patient who presented with a one-month history of severe frontal headache, followed by blurred vision and vomiting. On examination, he exhibited bilateral mydriatic pupils unresponsive to light, left eye outward deviation, ataxic gait, and asthenic appearance. Brain MRI revealed a large sellar mass suggestive of an invasive pituitary adenoma. Laboratory evaluation showed extreme hyperprolactinemia (50,260.9 ng/mL) and central hypothyroidism, leading to the diagnosis of a giant invasive prolactinoma. He was treated with cabergoline and levothyroxine. Due to neuropsychiatric symptoms (hallucinations, insomnia, and agitation), aripiprazole and melatonin were added. No evidence of apoplexy or intracranial hypertension was found. Craniotomy surgery was performed successfully. Clinical improvement was observed, but the patient was lost to follow-up. This case illustrates the classical presentation of giant prolactinoma in a male adolescent, emphasizing the size-prolactin correlation and the importance of early detection and medical treatment. Neuropsychiatric symptoms can emerge as a result of tumor mass effect or dopaminergic therapy. Long-term follow-up is essential to evaluate treatment response and prevent endocrine and developmental complications. Genetic testing for MEN1 or AIP mutations should be considered in young patients with aggressive pituitary adenomas.

## Introduction

Pituitary adenomas in childhood and adolescence are rare, accounting for only 0.1% to 0.3% of all intracranial tumors in the pediatric population [1]. Among these, prolactinomas are the most common functional subtype. Although they are more prevalent in the female population, male adolescents tend to present with larger and more aggressive tumors at diagnosis [2,3].

Clinical presentation varies according to the patient's sex and age. While females often present with amenorrhea and galactorrhea, males usually have delayed-onset symptoms dominated by headache (40%), visual impairment (60%), and signs of hypogonadism such as gynecomastia or pubertal arrest [4,5]. This pattern reflects the tendency for prolactinomas in males to grow significantly before detection, which explains the higher frequency of giant adenomas (>4 cm) in this population [6].

Despite favorable pharmacological outcomes in most cases, a subset of patients with invasive or medically resistant prolactinomas require surgical intervention, particularly in the presence of severe compressive symptoms such as progressive visual loss (60%) [7,8]. Currently, the endoscopic transsphenoidal approach is considered the preferred technique in specialized pediatric centers, with evidence supporting better visual outcomes and lower morbidity compared to conventional microsurgery [9].

## Case presentation

We present the case of a 15-year-old previously healthy male patient with a one-month history of intense frontal headache, described as incapacitating and partially relieved by paracetamol. The patient had no prior history of chronic illnesses, hospitalizations, or regular medication use. There was no reported history of trauma, infections, or visual problems prior to the onset of symptoms. At admission, the patient had not received any prescribed treatment apart from the occasional use of over-the-counter paracetamol (500 mg orally, as needed, no more than twice daily) for headache relief during the preceding week. No other medications, supplements, or hormonal therapies were used. The family history was non-contributory. There were no known cases of pituitary tumors, other intracranial neoplasms, or endocrine disorders among first- or second-degree relatives. Developmental history was unremarkable, with normal growth and pubertal milestones up to age 14. There was no history of gynecomastia, galactorrhea, or testicular abnormalities. No prior laboratory or imaging studies had been conducted before this admission, and no symptoms suggestive of prior hormonal imbalances-such as weight gain/loss, temperature intolerance, or fatigue-had been reported by the patient or his family.

**Figure 1:**
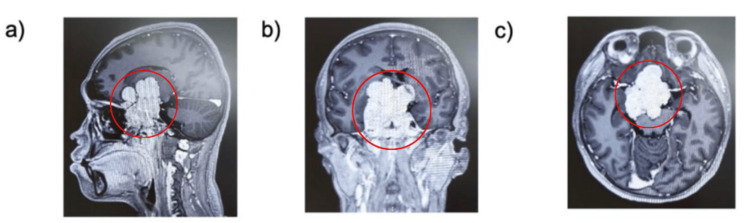
MRI demonstrating a giant invasive pituitary macroadenoma. Sagittal (a), coronal (b), and axial (c) T1-weighted post-contrast images reveal a large sellar and suprasellar mass with heterogeneous enhancement (circled in red). The tumor invades both cavernous sinuses, compresses the optic chiasm, and extends into adjacent structures

**Figure 2:**
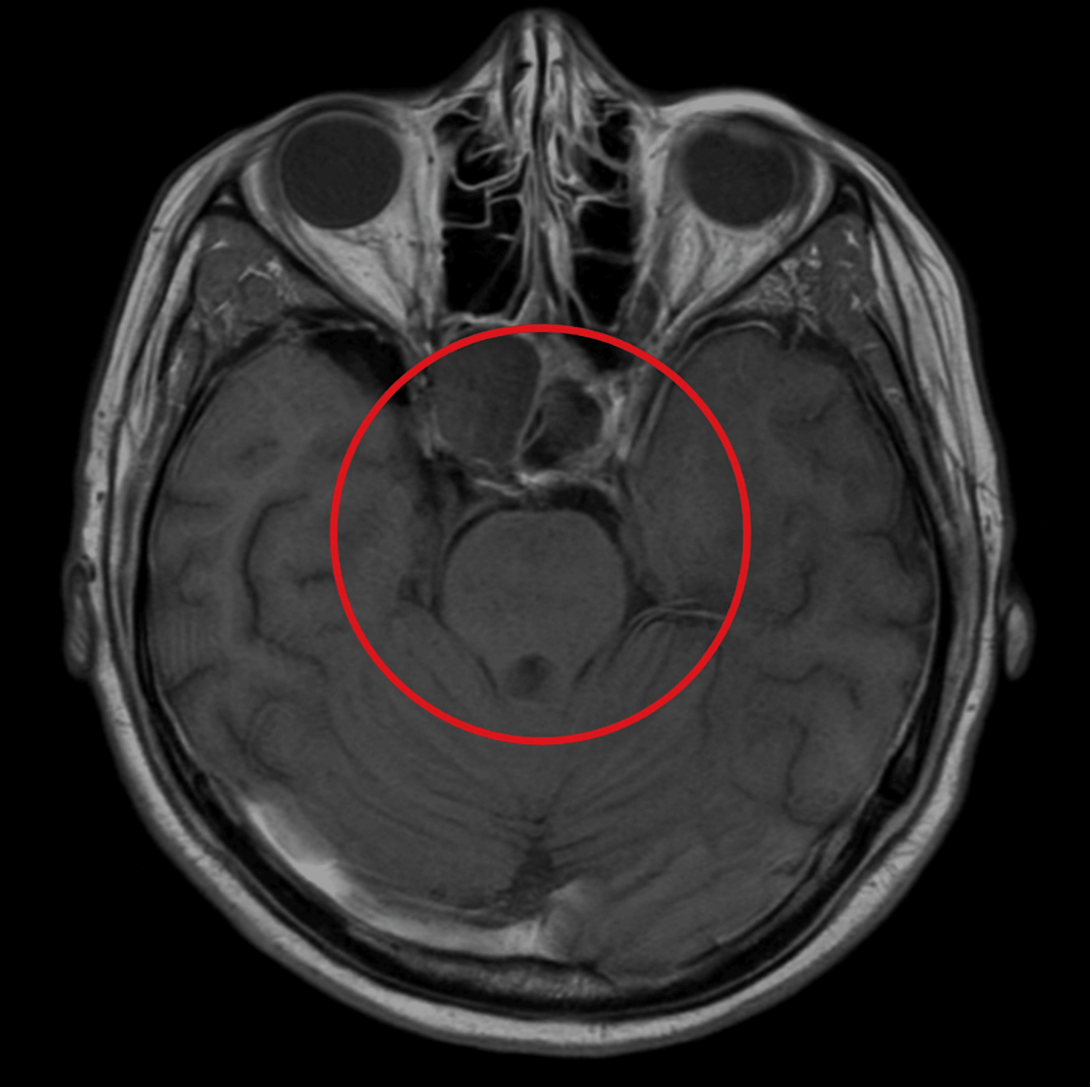
T1-weighted axial MRI showing a large, well-defined sellar and suprasellar mass (circled in red), consistent with an invasive pituitary macroadenoma. The lesion exerts a mass effect on the optic chiasm and extends laterally toward both cavernous sinuses

Over time, he developed blurred vision and a single episode of vomiting. On initial evaluation, the patient appeared pale and asthenic. Physical examination revealed bilateral mydriatic pupils unresponsive to light and outward deviation of the left eye, suggestive of cranial nerve involvement. He was unable to stand unassisted and exhibited an ataxic gait. Brain MRI (Figures 1, 2) revealed a large sellar mass consistent with an invasive pituitary adenoma. Laboratory results (Table 1) showed extreme hyperprolactinemia (50,260.9 ng/mL) and central hypothyroidism. A diagnosis of giant invasive prolactinoma was established. Although no histopathological confirmation was obtained, the diagnosis of giant prolactinoma was made based on the presence of markedly elevated prolactin (PRL) levels, characteristic MRI findings, and the exclusion of other potential causes of hyperprolactinemia.

**Table 1:** Patient's lab values during hospitalization TSH: thyroid-stimulating hormone; BUN: blood urea nitrogen

Parameter	Normal range	At admission	Pre-operative	Post-operative	At discharge
Prolactin	4.0-15.2 ng/mL	>50,000 ng/mL	>50,000 ng/mL	18,500 ng/mL	14,700 ng/mL
TSH	0.4-4.0 µIU/mL	0.01 µIU/mL	0.01 µIU/mL	0.25 µIU/mL	1.06 µIU/mL
Free T4	0.8-1.8 ng/dL	0.37 ng/dL	0.38 ng/dL	0.78 ng/dL	1.12 ng/dL
Glucose	70-100 mg/dL (fasting)	348 mg/dL	140 mg/dL	110 mg/dL	92 mg/dL
Hemoglobin	13.0-17.0 g/dL	15.1 g/dL	14.5 g/dL	13.7 g/dL	13.4 g/dL
Leukocytes	4,000-11,000/µL	17,450/µL	12,000/µL	10,400/µL	8,950/µL
Platelets	150,000-450,000/µL	317,000/µL	290,000/µL	270,000/µL	258,000/µL
Sodium	136-145 mEq/L	133.5 mEq/L	135 mEq/L	136.2 mEq/L	137.0 mEq/L
Potassium	3.5-5.0 mEq/L	4.9 mEq/L	4.5 mEq/L	4.3 mEq/L	4.1 mEq/L
Chloride	98-106 mEq/L	100.4 mEq/L	102 mEq/L	101.5 mEq/L	100.0 mEq/L
Creatinine	0.6-1.2 mg/dL	1.59 mg/dL	1.30 mg/dL	1.05 mg/dL	0.98 mg/dL
Urea	7-20 mg/dL	45.7 mg/dL	30.4 mg/dL	26.3 mg/dL	21.0 mg/dL
BUN	7-20 mg/dL	21.36 mg/dL	14.17 mg/dL	12.3 mg/dL	9.8 mg/dL

The patient was hospitalized for urgent evaluation by the neurosurgery and endocrinology teams. Medical treatment was initiated with cabergoline 0.5 mg orally, administered four times per week, along with levothyroxine 50 µg daily. Although some improvement in emotional and behavioral symptoms was noted with pharmacologic therapy, including aripiprazole and melatonin, a comprehensive evaluation of the tumor or hormonal response to cabergoline could not be completed due to the short follow-up and subsequent loss of the patient to outpatient care. Due to the development of neuropsychiatric symptoms (including auditory hallucinations, complete insomnia, agitation, and disorganized speech), aripiprazole was introduced at a dosage of 2.5 mg in the morning and 5 mg at night, based on a presumed diagnosis of mixed delirium or tumor-associated secondary psychosis. In addition, melatonin 5 mg at night was prescribed to aid sleep regulation, potentially disrupted by visual and dopaminergic dysfunction.

During hospitalization, no signs of intracranial hypertension or pituitary apoplexy were observed, and the surgical intervention was carried out without complications. The patient showed gradual clinical improvement, including enhanced emotional stability, partial restoration of sleep, and attenuation of psychotic symptoms. No interval imaging was performed prior to surgery to evaluate potential tumor shrinkage from cabergoline therapy. Thus, the impact of initial medical treatment on tumor size could not be assessed. He was discharged with instructions to continue medical therapy and to attend scheduled follow-up visits with endocrinology, neurosurgery, and psychiatry. Unfortunately, the patient was lost to follow-up, underscoring the critical importance of coordinated outpatient care and long-term monitoring in adolescent patients with pituitary tumors.

## Discussion

Pubertal development impairment is a key feature in these patients. Chronic hyperprolactinemia leads to hypogonadotropic hypogonadism, resulting in delayed or arrested puberty [3,10]. In the cohort by Breil et al. (12 patients, mean age 14 years), 67% presented with pubertal delay as the main clinical manifestation [10]. Similarly, Semple et al. reported a complete absence of secondary sexual characteristics in a 14-year-old male adolescent with a giant prolactinoma and a five-year history of symptoms [6]. Following successful treatment of the prolactinoma, normalization of the gonadal axis and appropriate pubertal development are typically achieved. In long-term follow-up, growth and puberty have been shown to resume normally after PRL control and tumor reduction [11]. In the series by Liu et al., which analyzed nine prepubertal children (<14 years) with surgically treated prolactinomas, growth and puberty evolved normally after treatment, reflecting endocrine recovery following pituitary decompression and PRL reduction [11]. This underscores the importance of timely diagnosis and treatment to prevent developmental sequelae.

On the other hand, systemic symptoms such as weight loss, asthenia, or growth retardation may also be present due to global pituitary dysfunction caused by compression. In our case, there was marked weight loss, possibly linked to central adrenal and thyroid insufficiency [6,7]. According to the literature, up to ~20% of children with prolactinoma may initially present with short stature or growth deceleration [6,12]. Webb and Prayson reported that four out of 20 pediatric patients had symptom onset before the age of 12, frequently with growth or developmental abnormalities, and that nearly all adenomas in children are secretory and clinically active [1]. Thus, the clinical presentation in male adolescents is often neurological (due to the mass effect of macroadenomas) and endocrine through pubertal hypogonadism; this profile aligns fully with the case presented.

Impairment of pubertal development is a hallmark of pediatric prolactinomas, resulting from chronic hyperprolactinemia-induced hypogonadotropic hypogonadism [3,10]. Breil et al. reported pubertal delay in 67% of their cohort (mean age 14 years), while Semple et al. described the complete absence of secondary sexual characteristics in a 14-year-old male patient with a giant prolactinoma and prolonged symptom history [6]. Following treatment, normalization of the gonadal axis and pubertal progression typically occurs. In the long term, growth and puberty resume after PRL reduction, as shown in a series of nine surgically treated prepubertal children reported by Liu et al. [11]. These findings underscore the importance of early diagnosis and intervention to prevent irreversible developmental consequences.

In prolactinomas, serum PRL concentration typically correlates with tumor burden. Microadenomas (≤10 mm) usually produce moderate PRL elevations, whereas macroadenomas (≥10 mm) reach significantly higher levels [2,3]. In the series by Acharya et al., the mean PRL at diagnosis was approximately 322 ng/mL in microadenomas and 522 ng/mL in macroadenomas, increasing to around 2,295 ng/mL in macroadenomas with suprasellar extension [2]. Similarly, Hoffmann et al. found that their pediatric macroadenomas (median volume 3.3 cm³) had median PRL levels of 890 ng/mL, while three “giant” tumors (>4 cm in diameter, volume ~44.5 cm³) had a median PRL of 4,720 ng/mL (range up to 10,400 ng/mL) [12]. Notably, the patient in the present case had a PRL level > 50,000 ng/mL, an extraordinarily high value that far exceeds the typical ranges reported even in giant prolactinomas. These levels suggest an immense functional tumor volume.

Quantitative studies confirm the correlation between tumor size and PRL levels. In the cohort by Yang et al. (25 patients ≤ 18 years), the maximum adenoma diameter showed a significant positive correlation with PRL levels at diagnosis (r = 0.710, p < 0.001) [4]. Similarly, the presence of panhypopituitarism-an indirect indicator of a large and invasive tumor-was associated with greater tumor size and higher PRL levels in that pediatric series [4]. Clinically, PRL values > 200 ng/mL are often considered suggestive of a macroprolactinoma until proven otherwise [7]. Kontbay et al., in a study of 27 children and adolescents with hyperprolactinemia, found that cases with prolactinoma had significantly higher PRL levels (median 118 ng/mL) than those with non-tumoral hyperprolactinemia (median ~39 ng/mL) [3]. Within the prolactinoma group, macroadenomas were associated with significantly higher PRL levels than microadenomas (median 200 ng/mL vs. 54.8 ng/mL; p = 0.006) and occurred at a younger age (median 13.8 vs. 17 years), with a maximum PRL level of 4,340 ng/mL in macroprolactinomas [3]. These findings suggest that larger lesions tend to present earlier due to more severe clinical manifestations.

Our case fits well within these observations: a 5 cm invasive tumor that produced a massive “supraphysiological” PRL level (>50,000 ng/mL). It is worth noting that such extreme levels can lead to systemic symptoms, such as the aforementioned weight loss due to secondary hypopituitarism. Kontbay et al. described a negative correlation between PRL levels and patients’ height standard deviation score (SDS) (r = -0.77), indicating that higher hyperprolactinemia is associated with greater growth impairment [3]. This suggests that excessive PRL (and the underlying tumor) impacts the growth axis, likely through central growth hormone or thyroid hormone deficiency. Overall, the evidence strongly supports a size-PRL correlation in pediatric prolactinomas: larger tumors secrete more hormones and cause greater dysfunction.

The management of macroprolactinomas in adolescents requires a multimodal and individualized approach, integrating medical therapy (dopamine agonists (DAs)) and, when necessary, transsphenoidal surgery. Clinical guidelines and case series consistently agree that the first-line treatment for prolactinomas-even in pediatric patients-is pharmacological, using DAs such as cabergoline or bromocriptine [2,3]. These drugs reduce PRL secretion and typically induce a marked decrease in tumor size in most cases, thereby avoiding the inherent risks of neurosurgical intervention.

In pediatric patients, cabergoline has proven to be highly effective and well tolerated. Breil et al. reported that cabergoline (initial dose ~0.5 mg/week) normalized PRL levels in eight out of 11 patients (73%) in their series, reduced tumor volume by an average of 80%, and led to clinical improvement in both headache and significant pubertal delay (p = 0.016 and p = 0.031, respectively) [10]. Although 55% of those patients experienced mild to moderate side effects with DAs, none required discontinuation of treatment [10]. In the patient presented in this case, cabergoline was initiated, which is consistent with these recommendations and with multiple reports of severe cases initially managed medically. In fact, there are reports of giant prolactinomas in adolescents treated exclusively with bromocriptine or cabergoline with excellent outcomes: Semple et al. described two male adolescents with blindness due to invasive macroprolactinomas who regained useful vision and achieved significant tumor reduction after several months of bromocriptine treatment, thereby avoiding surgery [6]. The patient in this case presented with bilateral amaurosis on admission-a critical situation in which cabergoline may have contributed rapidly to tumor mass reduction and potentially to some degree of initial visual improvement, although recovery ultimately depends on the extent of established axonal damage [6]. Nevertheless, transsphenoidal surgery plays a vital role in selected cases. Classic indications for surgery in prolactinomas include progressive visual deficits or pituitary apoplexy requiring urgent decompression, resistance or intolerance to DAs, and cases in which rapid symptomatic relief is desired due to massive tumor size [3,12].

It is important to emphasize that surgical outcomes in pediatric prolactinomas depend on tumor size and invasiveness. The surgical cure rate (remission without medication) is high in microprolactinomas but decreases significantly in invasive macroprolactinomas [4]. In the Korean series by Yang et al., only 53% of operated patients achieved normoprolactinemia after surgery [4]. Sinha et al. analyzed 12 children with giant adenomas (>40 mm) who underwent surgery: near-total resection (>90%) was achieved in 50% of cases, with visual improvement in 44%, but no patient with preoperative blindness recovered vision [8]. Surgical mortality in that series was 8% (one death), and 25% experienced severe perioperative complications [8]. Nevertheless, with multimodal therapy (surgery followed by radiotherapy and bromocriptine), all functional prolactinomas were in hormonal remission and had no progressive residual tumor at the last follow-up [8]. These data underscore that in massive prolactinomas, surgery alone is rarely curative; prolonged medical therapy and even radiotherapy are often required to control the residual tumor.

Regarding surgical technique, both endoscopic and microscopic transsphenoidal approaches are currently available. In specialized pediatric centers, the recent trend favors the endonasal endoscopic approach due to its broader visualization and reduced nasal invasiveness in young patients. Dhandapani et al. conducted a comparative study involving 48 patients aged ≤20 years who underwent surgery (33 with microscopy vs. 15 with endoscopy), finding that the rate of favorable outcomes (endocrine remission in functional tumors or total resection in non-functional ones) was significantly higher with the endoscopic technique (78.6% vs. 46.7%; OR = 4.18; p = 0.05) [9]. This better outcome with endoscopy was consistently observed across all age and tumor type subgroups, without an increase in complications [9]. Historical series using the conventional microscopic technique, such as that of Krajewski et al., also reported excellent results (88% hormonal remission in secreting adenomas) and no major surgical complications [13]. However, the incorporation of endoscopy appears to further optimize resection extent and neurovascular preservation in children. It is worth noting that in highly invasive adenomas (Hardy-Vézina 4, Knosp 3-4), complete removal is unlikely regardless of technique; surgery aims to relieve chiasmal compression and reduce tumor mass so that medical therapy (or radiotherapy) can control the residual lesion [8].

Visual recovery after chiasmal compression in giant prolactinomas is variable and depends on the extent of optic nerve damage. In Sinha et al.’s surgical series, 44% of children with giant adenomas experienced visual improvement postoperatively [8], whereas complete blindness (no light perception) was associated with poor recovery, as reported in both Semple et al.’s and Sinha et al.’s cases [6,9]. However, in patients with residual visual function, decompression-either surgical or medical-often leads to significant improvement. Notably, Semple et al. also described two adolescents with severe visual impairment who regained useful vision after bromocriptine treatment, highlighting the potential of medical therapy in select cases [6].

Regarding endocrine function, the goal is to normalize PRL levels and restore hormonal axes. With continuous treatment, biochemical remission (normal PRL without symptoms) is achieved in most cases. Andereggen et al. compared adolescents (<18 years) with older adults (≥65 years) with prolactinomas followed for ≥4 years and found remission in 90% of adolescents versus 70% of elderly patients, a difference that was not statistically significant [5]. Kontbay et al. reported that attempts to discontinue cabergoline after four years of successful treatment resulted in the recurrence of hyperprolactinemia in their adolescent patients, requiring the reintroduction of the drug [3]. This suggests that many pediatric prolactinomas require long-term pharmacologic treatment extending into adulthood to prevent recurrence. Adrenal function was not formally assessed during hospitalization, which represents a limitation, especially given the potential for central adrenal insufficiency in giant pituitary tumors. Although no signs of acute adrenal crisis were observed, biochemical evaluation (e.g., serum cortisol and adrenocorticotropic hormone (ACTH)) would have been essential for a complete assessment of pituitary function. Unfortunately, the patient's weight and nutritional assessment were not recorded at the time of admission, which limits the completeness of the clinical dataset presented.

In cases of giant prolactinomas with appropriate follow-up, long-term management involves serial monitoring of serum PRL levels (every 3-6 months initially), periodic pituitary MRI (typically at six months and then annually), and reassessment of pituitary hormonal axes. The titration of DA therapy is guided by both biochemical and radiological responses. Cabergoline is the preferred agent due to its higher efficacy and tolerability. In patients with persistent tumor mass or incomplete hormonal control, combination strategies including surgery or radiotherapy may be considered. Additionally, evaluation for germline mutations (e.g., AIP (aryl-hydrocarbon receptor-interacting protein) and MEN1 (menin gene)) should be discussed, particularly in adolescents or those with a family history suggestive of endocrine neoplasia syndromes. Long-term multidisciplinary care is essential to ensure endocrine recovery, visual function, and psychosocial development.

The early onset of pituitary adenomas raises the possibility of underlying genetic predisposition. Although most prolactinomas in children and adolescents are sporadic (not associated with hereditary syndromes) [14], a significant proportion may be linked to germline mutations in genes such as AIP or MEN1. van den Broek et al. conducted a systematic review on the relevance of genetic analysis in pituitary adenomas and concluded that AIP and MEN1 mutations are strongly associated with early-age presentation (e.g., gigantism or macroprolactinomas in adolescents) [14]. In fact, they recommend considering genetic testing in patients ≤ 30 years old with apparently sporadic pituitary adenomas [14]. AIP mutations tend to predispose to large, aggressive adenomas in young individuals-typically somatotropinomas, but also treatment-resistant prolactinomas. On the other hand, MEN1 syndrome (multiple endocrine neoplasia type 1) can manifest during adolescence with prolactinoma in combination with hyperparathyroidism or pancreatic tumors; however, in the absence of other endocrine findings, MEN1 is less likely.

## Conclusions

This case of a giant invasive macroprolactinoma in a 15-year-old male patient exemplifies the clinical, surgical, and endocrine challenges inherent to pediatric prolactinomas. The presentation with amaurosis, severe headache, and gynecomastia reflected the typically aggressive behavior observed in male adolescents, where extreme hyperprolactinemia correlates with a high tumor burden and profound pituitary dysfunction. Treatment with cabergoline followed by partial transsphenoidal surgery achieved clinical and biochemical stabilization, consistent with evidence supporting this combined approach as the most effective for invasive macroprolactinomas-demonstrating sustained hormonal control in most cases and surgical relief when mass effect is significant.

Follow-up should aim to maintain normoprolactinemia, monitor the residual tumor via imaging (ideally with MRI at six months after initiating treatment, then annually if stable), and assess serum PRL levels every three to six months during the first year, then biannually thereafter. Hormonal axes should be reevaluated at least yearly or if symptoms suggest new endocrine dysfunction. This case aligns with reports highlighting greater tumor aggressiveness in male adolescents, the efficacy of DAs as first-line therapy, and the role of transsphenoidal resection even in giant adenomas. Furthermore, the importance of excluding genetic etiologies such as AIP mutations or MEN1 syndrome in these patients cannot be overstated. Therapeutic success in such cases requires a continuous multidisciplinary approach to ensure appropriate neuroendocrine development and long-term quality of life.
